# Prevalence, predictors, and patient-reported outcomes of long COVID in hospitalized and non-hospitalized patients from the city of São Paulo, Brazil

**DOI:** 10.3389/fpubh.2023.1302669

**Published:** 2024-01-22

**Authors:** Daniel Tavares Malheiro, Sabrina Bernardez-Pereira, Kauê Capellato Junqueira Parreira, João Gabriel Dias Pagliuso, Emerson de Paula Gomes, Daisa de Mesquita Escobosa, Carolina Ivo de Araújo, Beatriz Silva Pimenta, Vivian Lin, Silvana Maria de Almeida, Paula Tuma, Claudia Regina Laselva, Miguel Cendoroglo Neto, Sidney Klajner, Vanessa Damazio Teich, Takaaki Kobayashi, Michael B. Edmond, Alexandre R. Marra

**Affiliations:** ^1^Hospital Israelita Albert Einstein, São Paulo, Brazil; ^2^Department of Internal Medicine, University of Iowa Carver College of Medicine, Iowa City, IA, United States; ^3^West Virginia University School of Medicine, Morgantown, WV, United States

**Keywords:** long COVID, mental health, depression screening, quality of life, middle income countries

## Abstract

**Background:**

Robust data comparing long COVID in hospitalized and non-hospitalized patients in middle-income countries are limited.

**Methods:**

A retrospective cohort study was conducted in Brazil, including hospitalized and non-hospitalized patients. Long COVID was diagnosed at 90-day follow-up using WHO criteria. Demographic and clinical information, including the depression screening scale (PHQ-2) at day 30, was compared between the groups. If the PHQ-2 score is 3 or greater, major depressive disorder is likely. Logistic regression analysis identified predictors and protective factors for long COVID.

**Results:**

A total of 291 hospitalized and 1,118 non-hospitalized patients with COVID-19 were included. The prevalence of long COVID was 47.1% and 49.5%, respectively. Multivariable logistic regression showed female sex (odds ratio [OR] = 4.50, 95% confidence interval (CI) 2.51–8.37), hypertension (OR = 2.90, 95% CI 1.52–5.69), PHQ-2 > 3 (OR = 6.50, 95% CI 1.68–33.4) and corticosteroid use during hospital stay (OR = 2.43, 95% CI 1.20–5.04) as predictors of long COVID in hospitalized patients, while female sex (OR = 2.52, 95% CI 1.95–3.27) and PHQ-2 > 3 (OR = 3.88, 95% CI 2.52–6.16) were predictors in non-hospitalized patients.

**Conclusion:**

Long COVID was prevalent in both groups. Positive depression screening at day 30 post-infection can predict long COVID. Early screening of depression helps health staff to identify patients at a higher risk of long COVID, allowing an early diagnosis of the condition.

## 1 Introduction

The World Health Organization (WHO) defines long COVID as the continuation or development of new symptoms 3 months after the initial SARS-CoV-2 infection, with these symptoms lasting for at least 2 months with no other explanation. Long COVID impacts various population groups, leading to a diverse array of signs and symptoms. Over 200 different symptoms have been reported that can have an impact on daily life activities (1). It poses a growing medical challenge due to the complexity and diversity of its long-term effects. The presence of respiratory, motor, cardiovascular, or psychological sequelae heightens the demand for physical rehabilitation services and psychosocial support. Consequently, long COVID is increasingly burdening the healthcare system. Apart from strengthening the primary healthcare system and its multidisciplinary teams, there is a need to enhance specialized care. Considering that middle-income countries may have limited access to healthcare systems with fewer resources compared to high-income countries, it is crucial to investigate the prevalence of long COVID in these middle-income countries.

The majority of studies on long COVID have been carried out in Europe and North America, focusing on patients who were hospitalized and later discharged (2, 3). The United States produced the largest number of related publications, followed by the United Kingdom. The top ten most frequent keywords cited in these publications are “fatigue,” “depression,” and “inflammation” (2). Long COVID appears to be more common among women, older adult individuals, and those with existing comorbidities and higher body mass index (BMI) (2). However, limited research has been conducted on the long-term predictors in patients from middle-income countries comparing hospitalized and non-hospitalized patients. A systematic literature review published in 2021 showed that the number of publications had the following geographic distribution: Europe (62%, 24/39), followed by Asia (23%, 9/39), North America (8%, 3/39) and the Middle East 8% (3/39) and none of the included studies were carried out in low-middle-income countries (4). Furthermore, another systematic literature review and meta-analysis comprising 139 studies, highlighted a limitation of the geographic homogeneity. Over 50% of publications originated from Europe, with, less than 5% representing studies in long COVID from Africa, Oceania and South America. This underscores the need for more data from low-middle income countries (5). The study postulates that the prevalence of long COVID may exhibit variations between hospitalized and non-hospitalized COVID-19 patients in middle-income countries. Additionally, specific demographic and clinical factors, including patient-reported outcomes variables, including aspects of mental health, may serve as predictors of long COVID. The relationship between depression and long COVID is still an ongoing area of research and results are not conclusive.

The primary objective of this research is to assess the prevalence of long COVID among both hospitalized and non-hospitalized patient cohorts. Furthermore, the study seeks to identify predictive and protective factors influencing the development of long COVID within these groups. This investigation also endeavors to evaluate the impact of long COVID on the quality of life among afflicted individuals in Brazil.

## 2 Materials and methods

### 2.1 Population under study, study design, criteria of eligibility

This was a single center, retrospective cohort study, which included all the hospitalized and non-hospitalized adult patients (with a confirmed diagnosis of COVID-19 at Hospital Israelita Albert Einstein (HIAE) from February 12, 2021 to July 25, 2022. The Brazilian Israelite Society Albert Einstein is a nonprofit healthcare, educational, and research organization, with headquarters in the city of São Paulo, managing diverse services from primary to tertiary care, in the public and private healthcare sectors. It operates 40 healthcare units, mainly in the state of São Paulo. In 2022, the private sector HIAE had 344,000 emergency department visits, 495,000 outpatient visits, and 62,000 hospital discharges. The institution manages a diverse healthcare system ranging from primary healthcare to tertiary care services in the public and private sectors.

Any hospitalized and non-hospitalized adult patients (18 or more years of age) with a laboratory-confirmed COVID-19 were included. The laboratorial confirmation was performed using RT-PCR on specimens obtained via naso-pharyngeal swab, according to the protocol instituted at HIAE.

### 2.2 Long COVID definition

Long COVID was defined according to WHO criteria as the continuation or development of new symptoms 3 months after the initial SARS-CoV-2 infection. The signs and symptoms included in long COVID were general signs and symptoms (fatigue), respiratory and cardiac symptoms (dyspnea, cough, chest pain), neurological symptoms (memory loss, headache, sleep problem), and other symptoms (anosmia, ageusia, motor problems and difficulties with activities of daily living). We collected data on the first SARS-CoV-2 infection recorded in our system and excluded patients who were subsequently diagnosed with re-infection documented in the electronic medical record.

### 2.3 Follow Up

All the hospitalized patients with COVID-19 were followed through telephone interviews run by a trained professional 30 days and 90 days after hospital discharge, If the subject was unreachable at first call, three attempts were made. Non-hospitalized patients were followed 30 days and 90 days after COVID-19 confirmation date, via text message or email.

### 2.4 Data collection and measures

Data were collected using an electronic medical record, and patient reported questionnaires. At baseline, demographic characteristics including age, sex, and BMI, and clinical information including symptoms on admission, disease duration from onset of symptoms and underlying comorbidities were collected. Intensive care unit admission, use of mechanical ventilation, length of hospital stay, and drug therapy (i.e., steroids, antibiotics, and remdesivir) were also collected for hospitalized patients.

At 30-day and 90-day follow-up, symptoms and the PHQ-2 questionnaire were collected in both groups, and the EuroQol-5D3L quality of life questionnaire and EuroQol visual analog scale (EQ-VAS) were collected only in the hospitalized patient group.

#### 2.4.1 Structured questionnaires

The EuroQol-5D3L is a generic instrument for measuring health-related quality of life which generates an EQ-5D index score from 1 (full health) to 0 (a state as bad as being dead). The EQ-VAS is a 0–100 scale where patients are asked to indicate their overall health, where the higher the value, the better. Patients with and without long COVID were analyzed according to change in the EQ-5D index score and EQ-VAS scale from 30 days to 90 days. Three categories were defined: improvement, no change and worsening (6).

The PHQ-2 addresses the frequency of depressed mood and anhedonia in the last two weeks and can be used as a first approach for diagnosing depression. If the score is 3 or greater, major depressive disorder is likely (7).

### 2.5 Period of COVID-19 variants

As only a small number of positive samples among our cases were sequenced, all individuals were classified according to the most prevalent variant. Due to the low number of Alpha cases, it was combined with the Gamma cases to form a single time period. The time period between February 12, 2021, to August 5, 2021 was considered the “Alpha/Gamma era”; August 6, 2021 to December 16, 2021 the “Delta era”; and December 17, 2021 to July 25, 2022, the “Omicron era” (8).

### 2.6 Statistical analysis

To compare the demographic characteristics of hospitalized and non-hospitalized patients among COVID-19 variant eras, the Pearson’s Chi-square and Fisher’s exact test were used for categorical variables and were summarized as counts and percentages (9). In addition, normality assumptions were tested by the Anderson-Darling normality test. If this test provided evidence against a normal distribution for a given continuous variable, the Wilcoxon-Mann–Whitney U test was used instead. Both were expressed as medians with IQR (Interquartile Range).

A logistic regression model was used to investigate which factor, either at baseline or follow-up day 30, was associated with long COVID at day 90 in the two groups (hospitalized and non-hospitalized patients). The variables selected to enter the multivariate model were those with significant associations on univariate analysis (*p* < 0.05). Dichotomous intervals for continuous variables such as age (≥60 years or < 60 years) and length of stay (≥21 days or < 21 days) were created for the models. For the purposes of logistic regression, the PHQ-2 response on day 30 was used. Some variables did not have full information for all observations, for instance PHQ2 ≥ 3 (n = 6) and obesity (n = 4) had a total of 281 patients (long COVID = 131 and no long COVID = 150) and these variables were removed from the hospitalized long COVID prediction model. Predictors for both models did not present variance inflation factors (VIF >10), indicating that collinearity was not a problem. Receiver Operating Characteristic (ROC) curve analysis was employed to evaluate the performance of the predictive model. The Area Under the ROC Curve (AUC) was calculated to assess the discriminatory ability of the model in distinguishing between long COVID and no long COVID. All results with *p* < 0.05 were considered statistically significant. Data was manipulated in Knime Analytics Software^1^ and all statistical analyses were performed in R (4.2.0 version)^2^ programming language. The R packages used in the analyses are described in Supplementary Data 1.

### 2.7 Ethics approval

The study was approved by the HIAE Research Ethics Committee, protocol number 6.204.804, CAAE: 69689123.2.0000.0071, and the National Commission for Research Ethics.

## 3 Results

### 3.1 Demographic and clinical characteristics of hospitalized and non-hospitalized patients

**Figure 1:**
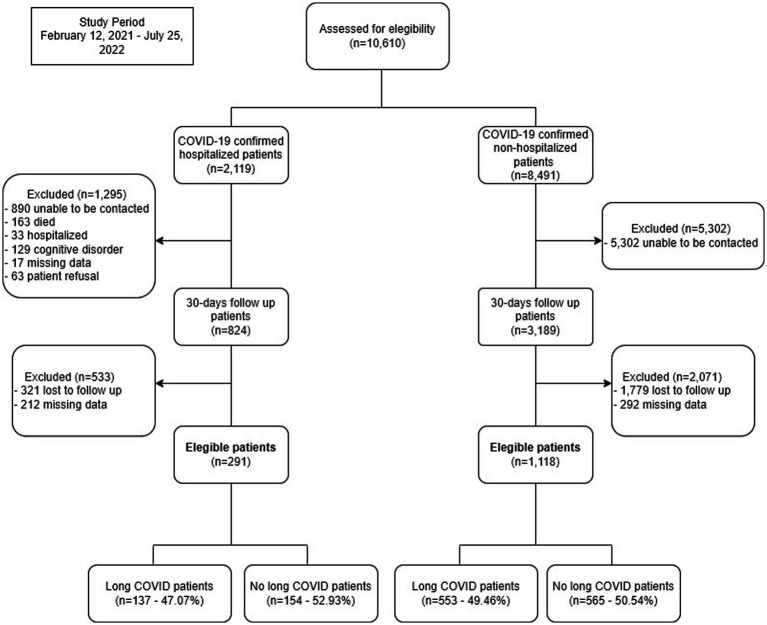
Study flowchart.

During the study period, 1,409 patients with confirmed COVID-19 were included, of which 291 (20.65%) were hospitalized patients and 1,118 (79.35%) non-hospitalized patients (Figure 1).

Table 1 summarizes the demographic and clinical characteristics of the eligible population in the two study groups (hospitalized and non-hospitalized) stratified by COVID-19 eras. Patients who were hospitalized were more likely to be older (median age 53.0 vs. 43.0 years old, *p* < 0.01) than non-hospitalized patients, regardless of variant, and had higher BMI (27.9 vs. 25.7 kg/m^2^, *p* < 0.01) in the Alpha/Gamma era. Hospitalized patients also had more comorbidities and the predominant symptoms on admission were cough (35.7% vs. 28.2%, *p* = 0.01), fever (55.7% vs. 20.5%, *p* < 0.01), myalgia (30.6% vs. 19.7%, *p* < 0.01), fatigue (32.0% vs. 25.1%, *p* = 0.02) and dyspnea (19.9% vs. 5.4%, *p* < 0.01) when compared to non-hospitalized patients. Regarding the clinical course among hospitalized patients, 42.3% required admission to the ICU, 9.6% received mechanical ventilation, 59.5% antibiotics, 69.8% steroids and 12.4% remdesivir. Patients admitted during the Alpha/Gamma era received proportionally more mechanical ventilation (14.6%, *p* = 0.01) and corticosteroids (93.4%, *p* < 0.01) (Supplementary Table 1).

**Table 1:** Demographic and clinical characteristics of the eligible population by COVID-19 era.

Characteristic	Overall			Alpha/Gama			Delta			Omicron		
	Hospitalized N = 291	Non-Hospitalized N = 1,118	*p*-value^*^	Hospitalized N = 151	Non-Hospitalized N = 303	*p*-value*^†^*	Hospitalized, N = 15	Non-Hospitalized N = 38	*p*-value*^‡^*	Hospitalized N = 125	Non-Hospitalized N = 777	*p*-value^*^
Sex, n (%)												
Male	187 (64.26%)	427 (38.19%)	**<0.01**	107 (70.86%)	127 (41.91%)	**<0.01**	6 (40.00%)	17 (44.74%)	0.75	74 (59.20%)	283 (36.42%)	**<0.01**
Age, Median (P25-P75)	53.00 (43.00–67.00)	43.00 (36.00–53.00)	**<0.01**	47.00 (40.00–55.00)	43.00 (37.00–53.00)	**<0.01**	59.00 (42.00–73.00)	42.73 (38.41–49.41)	**<0.01**	66.00 (51.00–77.00)	42.82 (35.53–53.50)	**<0.01**
BMI, Median (P25-P75)*^§^*	27.31 (24.72–29.98)	25.99 (23.31–29.40)	**<0.01**	27.85 (25.62–30.94)	25.72 (23.41–29.39)	**<0.01**	24.92 (22.89–28.09)	26.84 (24.97–30.21)	0.15	26.30 (24.01–29.39)	25.99 (23.23–29.40)	0.42
Missing	4	461		0	211		1	16		3	234	
Rhinorrhea, n (%)	52 (17.87%)	308 (27.55%)	**<0.01**	24 (15.89%)	47 (15.51%)	0.92	1 (6.67%)	10 (26.32%)	0.15	27 (21.60%)	251 (32.30%)	**0.02**
Cough, n (%)	104 (35.74%)	315 (28.18%)	**0.01**	51 (33.77%)	48 (15.84%)	**<0.01**	8 (53.33%)	9 (23.68%)	0.05	45 (36.00%)	258 (33.20%)	0.54
Fever, n (%)	162 (55.67%)	229 (20.48%)	**<0.01**	94 (62.25%)	38 (12.54%)	**<0.01**	6 (40.00%)	10 (26.32%)	0.34	62 (49.60%)	181 (23.29%)	**<0.01**
Sore Throat, (%)	60 (20.62%)	344 (30.77%)	**<0.01**	18 (11.92%)	36 (11.88%)	0.99	0 (0.00%)	9 (23.68%)	**0.05**	42 (33.60%)	299 (38.48%)	0.30
Myalgia, (%)	89 (30.58%)	220 (19.68%)	**<0.01**	53 (35.10%)	37 (12.21%)	**<0.01**	0 (0.00%)	7 (18.42%)	0.17	36 (28.80%)	176 (22.65%)	0.13
Headache, n (%)	62 (21.31%)	267 (23.88%)	0.35	36 (23.84%)	49 (16.17%)	**0.05**	4 (26.67%)	11 (28.95%)	1.00	22 (17.60%)	207 (26.64%)	**0.03**
Fatigue, n (%)	93 (31.96%)	281 (25.13%)	**0.02**	45 (29.80%)	49 (16.17%)	**<0.01**	6 (40.00%)	10 (26.32%)	0.34	42 (33.60%)	222 (28.57%)	0.25
Dyspnea, n (%)	58 (19.93%)	60 (5.37%)	**<0.01**	30 (19.87%)	16 (5.28%)	**<0.01**	4 (26.67%)	1 (2.63%)	**0.02**	24 (19.20%)	43 (5.53%)	**<0.01**
Anosmia, n (%)	16 (5.50%)	120 (10.73%)	**<0.01**	9 (5.96%)	45 (14.85%)	**<0.01**	1 (6.67%)	17 (44.74%)	**<0.01**	6 (4.80%)	58 (7.46%)	0.28
Dysguesia, n (%)	18 (6.19%)	102 (9.12%)	0.11	10 (6.62%)	37 (12.21%)	0.07	1 (6.67%)	14 (36.84%)	**0.04**	7 (5.60%)	51 (6.56%)	0.68
Nausea, n (%)	28 (9.62%)	55 (4.92%)	**<0.01**	9 (5.96%)	10 (3.30%)	0.18	1 (6.67%)	5 (13.16%)	0.66	18 (14.40%)	40 (5.15%)	**<0.01**
Diarrhea, n (%)	6 (2.06%)	90 (8.05%)	**<0.01**	2 (1.32%)	15 (4.95%)	0.06	0 (0.00%)	4 (10.53%)	0.57	4 (3.20%)	71 (9.14%)	**0.03**
Hypertension, n (%)	74 (25.43%)	90 (8.05%)	**<0.01**	32 (21.19%)	11 (3.63%)	**<0.01**	3 (20.00%)	2 (5.26%)	0.13	39 (31.20%)	77 (9.91%)	**<0.01**
Diabetes, n (%)	46 (15.81%)	37 (3.31%)	**<0.01**	15 (9.93%)	4 (1.32%)	**<0.01**	1 (6.67%)	0 (0.00%)	0.28	30 (24.00%)	33 (4.25%)	**<0.01**
Obesity, n (%)	71 (24.74%)	153 (23.29%)	0.63	47 (31.13%)	22 (23.91%)	0.23	1 (7.14%)	6 (27.27%)	0.21	23 (18.85%)	125 (23.02%)	0.32
Missing	4	461		0	211		1	16		3	234	
COPD, n (%)^||^	19 (6.53%)	29 (2.59%)	**<0.01**	6 (3.97%)	5 (1.65%)	0.19	1 (6.67%)	0 (0.00%)	0.28	12 (9.60%)	24 (3.09%)	**<0.01**
Cancer, n (%)	8 (2.75%)	5 (0.45%)	**<0.01**	0 (0.00%)	0 (0.00%)		0 (0.00%)	0 (0.00%)		8 (6.40%)	5 (0.64%)	**<0.01**

*Pearson’s Chi-squared test; Wilcoxon rank sum test; Fisher’s exact test. ^†^Pearson’s Chi-squared test; Wilcoxon rank sum test; Welch Two Sample t-test; Fisher’s exact test. ^‡^ Pearson’s Chi-squared test; Wilcoxon rank sum exact test; Wilcoxon rank sum test; Fisher’s exact test. ^§^ BMI = Body Mass Index. ^‖^ COPD = Chronic Obstructive Pulmonary Disease. * Pearson’s Chi squared test; Wilcoxon rank sum test; Fisher’s exact test | † Pearson’s Chi-squared test; Wilcoxon rank sum test; Welch Two Sample t-test; Fisher’s exact test | ‡ Pearson’s Chi-squared test; Wilcoxon rank sum exact test; Wilcoxon rank sum test; Fisher’s exact test | § BMI = Body Mass Index | ‖ COPD = Chronic Obstructive Pulmonary Disease. Values in bold are statistically significant (*p* < 0.05).

### 3.2 Long COVID prevalence and its characteristics

The prevalence of long COVID was found to be 47.1% among hospitalized patients and 49.5% among non-hospitalized patients at 90 days. Among hospitalized patients, the prevalence of long COVID varied across different eras, with rates of 58.3% during the Alpha/Gamma era, 46.7% during the Delta era, and 33.6% during the Omicron era. Among non-hospitalized patients, the prevalence of long COVID was 50.83% during the Alpha/Gamma era, 63.16% during the Delta era, and 48.26% during the Omicron era. In hospitalized patients diagnosed with long COVID, the most common symptoms reported were memory loss (33.6%) and fatigue (30.7%). Among non-hospitalized patients, the prevalent symptoms included memory loss (45.8%), fatigue (46.5%), sleep disorders (32.4%) and headache (30.7%) (Supplementary Figure 1 provides further details).

**Figure 2:**
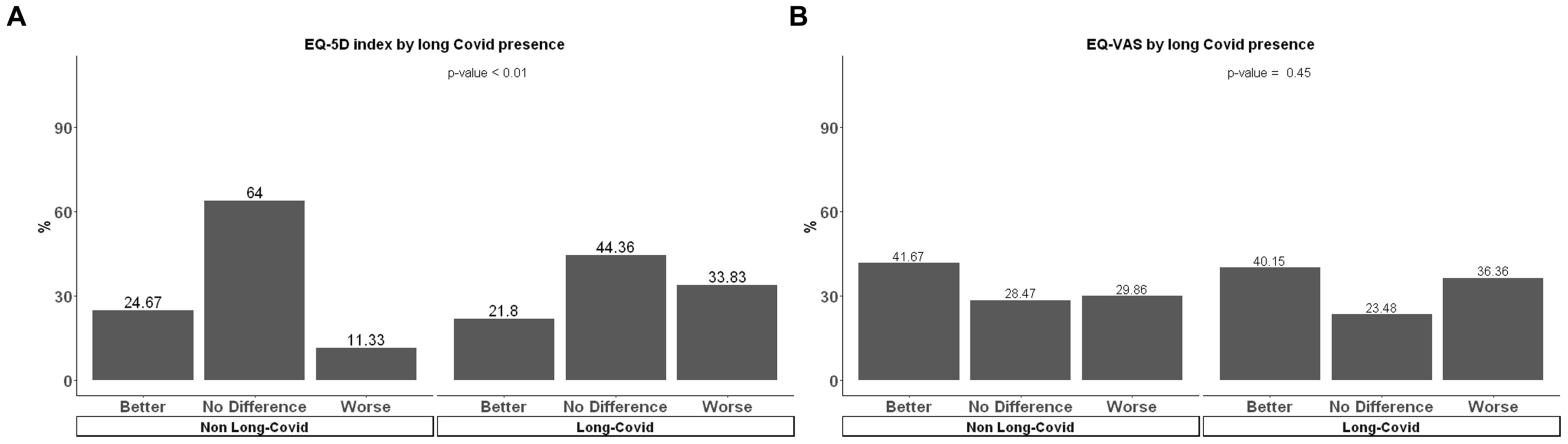
**(A)** EQ-5D index and **(B)** EQ-VAS change from 30 days to 90 days follow up for long COVID and no long COVID of hospitalized patients.

The analysis of the change in the EQ-5D index score from 30 days to 90 days showed that patients with long COVID have a higher rate of worsening over time than those without long COVID (33.8% vs. 11.3%, *p* < 0.01) (Figure 2A). There was no significant difference for the EQ-VAS variation score in the same period (*p* = 0.45) (Figure 2B), however, the EQ-VAS score at 90 days was lower among patients with long COVID compared to those without this condition (Supplementary Figure 2).

### 3.3 Predictive and protective factors of long COVID

Multivariable logistic regression showed that predictors of long COVID among hospitalized patients were female sex (odds ratio [OR] = 4.50, 95% confidence interval (CI): [2.51–8.37]), hypertension (OR = 2.90 [1.52–5.69]), PHQ-2 ≥ 3 (OR = 6.50 [1.68–33.4]) and corticosteroid treatment during hospital stay (OR = 2.43 [1.20–5.04]) and a protective factor was Omicron era (OR = 0.40 [0.19–0.83]) (Table 2). Among non-hospitalized patients, predictors were female sex (OR = 2.52 [1.95–3.27]) and PHQ-2 > 3 (OR = 3.88 [2.52–6.16]) and a protective factor was age ≥ 60 years old (OR = 0.68 [0.48–0.97]) (Table 3).

**Table 2:** Multivariate analysis of predictors for long COVID in hospitalized patients.

Characteristic	No longCOVID, N = 150	Long COVID, N = 131	Univariate analysis		Multivariate analysis	
			OR (95% CI)^*^	*p*-value	OR (95% CI)^*^	*p*-value
Era, n (%)						
Alpha/Gamma	63 (42.00)	87 (66.41)	—		—	
Delta	8 (5.33)	6 (4.58)	0.54 (0.17–1.64)	0.28	0.57 (0.15–2.01)	0.38
Omicron	79 (52.67)	38 (29.01)	0.35 (0.21–0.57)	**<0.01**	0.40 (0.19–0.83)	**0.01**
Sex, n (%)						
Female	38 (25.33)	64 (48.85)	2.82 (1.71–4.69)	**<0.01**	4.50 (2.51–8.37)	**<0.01**
Age, n (%)						
> = 60 years	62 (41.33)	36 (27.48)	0.54 (0.32–0.89)	**0.02**	0.59 (0.30–1.15)	0.12
ALOS, n (%)*^†^*						
> = 21 days	15 (10.00)	17 (12.98)	1.34 (0.64–2.84)	0.43		
Intensive Care Unit, n (%)						
Yes	63 (42.00)	54 (41.22)	0.97 (0.60–1.56)	0.89		
Mechanical ventilation, n (%)						
Yes	13 (8.67)	14 (10.69)	1.26 (0.57–2.82)	0.57		
Hypertension, n (%)						
Yes	31 (20.67)	41 (31.30)	1.75 (1.02–3.02)	**0.04**	2.90 (1.52–5.69)	**<0.01**
Diabetes, n (%)						
Yes	27 (18.00)	17 (12.98)	0.68 (0.35–1.30)	0.25		
COPD, n (%)*^‡^*						
Yes	12 (8.00)	7 (5.34)	0.65 (0.24–1.67)	0.38		
CKD, n (%)*^§^*						
Yes	7 (4.67)	4 (3.05)	0.64 (0.17–2.18)	0.49		
Cancer, n (%)						
Yes	5 (3.33)	2 (1.53)	0.45 (0.06–2.13)	0.34		
Hypothyroidism, n (%)						
Yes	6 (4.00)	7 (5.34)	1.35 (0.44–4.31)	0.59		
Obesity, n (%)						
Yes	31 (20.67)	39 (29.77)	1.63 (0.95–2.82)	0.08		
Dyslipidemia, n (%)						
Yes	8 (5.33)	12 (9.16)	1.79 (0.72–4.71)	0.22		
PHQ2 ≥ 3, n (%)						
Yes	3 (2.00)	12 (9.16)	4.94 (1.53–22.1)	**0.02**	6.50 (1.68–33.4)	**0.01**
Remdesivir, n (%)						
Yes	20 (13.33)	14 (10.69)	0.78 (0.37–1.60)	0.50		
Antibiotics, n (%)						
Yes	82 (54.67)	83 (63.36)	1.43 (0.89–2.32)	0.14		
Corticosteroids, n (%)						
Yes	90 (60.00)	107 (81.68)	2.97 (1.73–5.22)	**<0.01**	2.43 (1.20–5.04)	**0.02**

*OR = Odds Ratio, CI = Confidence Interval. ^†^ALOS = Average length of stay. ^‡^ COPD = Chronic Obstructive Pulmonary Disease. ^§^CKD = Chronic Kidney Disease. Values in bold are statistically significant (*p* < 0.05).

**Table 3:** Multivariate analysis of predictors for long COVID in non-hospitalized patients.

Characteristic	No long COVID, N = 565	Long COVID, N = 553	Univariate analysis		Multivariate analysis	
			OR (95% CI)^*^	*p*-value	OR (95% CI)^*^	*p*-value
Era, n (%)						
Alpha/Gamma	149 (26.37)	154 (27.85)	—			
Delta	14 (2.48)	24 (4.34)	1.66 (0.84–3.40)	0.15		
Omicron	402 (71.15)	375 (67.81)	0.90 (0.69–1.18)	0.45		
Sex, n (%)						
Female	283 (50.09)	408 (73.78)	2.80 (2.18–3.61)	**<0.01**	2.52 (1.95–3.27)	**<0.01**
Age, n (%)						
≥60 years	109 (19.29)	60 (10.85)	0.51 (0.36–0.71)	**<0.01**	0.68 (0.48–0.97)	**0.04**
Hypertension, n (%)						
Yes	49 (8.67)	41 (7.41)	0.84 (0.55–1.30)	0.44		
Diabetes, n (%)						
Yes	15 (2.65)	22 (3.98)	1.52 (0.79–3.02)	0.22		
COPD, n (%)*^†^*						
Yes	13 (2.30)	16 (2.89)	1.27 (0.60–2.70)	0.53		
CKD, n (%)*^‡^*						
Yes	2 (0.35)	3 (0.54)	1.54 (0.25–11.7)	0.64		
Cancer, n (%)						
Yes	4 (0.71)	1 (0.18)	0.25 (0.01–1.72)	0.22		
PHQ2 ≥ 3, n (%)						
Yes	28 (4.96)	105 (18.99)	4.49 (2.95–7.07)	**<0.01**	3.88 (2.52–6.16)	**<0.01**

*OR = Odds Ratio, CI = Confidence Interval. ^†^COPD = Chronic Obstructive Pulmonary Disease. ^‡^CKD – Chronic Kidney Disease. Values in bold are statistically significant (*p* < 0.05).

The ROC curve was employed to analyze the predictive power of the variables for the classification of long COVID. For the group of hospitalized patients, the analyzed variables were gender, age (> = 60 years), hypertension, PHQ2, and corticosteroid use, resulting in an area under the curve (AUC) value of 0.759 and a 95% confidence interval between 0.704 and 0.815. Regarding the group of outpatient patients, the available variables were gender, age (> = 60 years), and PHQ2, yielding an AUC value of 0.667 and a 95% confidence interval between 0.638 and 0.696 (Supplementary Figures 3, 4).

## 4 Discussion

This study revealed that the prevalence of long COVID among hospitalized patients and non-hospitalized patients was 47.1 and 49.5%, respectively, at the 90 days after initial SARS-CoV-2 infection in a middle-income country. The prevalence of long COVID varied across different variant eras with the lowest prevalence seen in the Omicron era. Memory loss and fatigue were the most common symptoms for both groups. The factors associated with long COVID were female gender and positive screening for depression (PHQ-2 score) in both groups. The presence of hypertension also showed a risk for the development of long COVID among hospitalized patients while Omicron era infection was associated with a lower risk. Additionally, hospitalized patients with long COVID had a higher percentage of worsening quality of life measured by the EQ-5D index score at 90 days when compared to patients without long COVID.

A surveillance report of the European Centre for Disease Prevention and Control (ECDC), through a systematic review study and meta-analysis revealed a higher incidence of long COVID in patients admitted to the ICU. In this report overall prevalence of any post COVID-19 condition symptom was estimated at 51% in the community setting; 67% in the hospital setting; and 74% in the ICU setting (3). More recently, another systematic review signaled that the prevalence estimates of long COVID were significantly influenced by the severity of acute infection and being hospitalized (10). However, our results showed similar overall prevalence among non-hospitalized patients and hospitalized patients.

Interestingly, our study showed that the prevalence of long COVID was lowest during the Omicron era in hospitalized patients. A recent systematic review demonstrated that patients infected with the Omicron variant may have a lower risk of developing long COVID than those infected with other variants (11). However, the Omicron era was not shown to be a protective factor in non-hospitalized patients and further research is needed to understand the specific mechanisms underlying this observation and to determine if it holds true across different populations and settings.

Limited research exists on long COVID among hospitalized and non-hospitalized patients in middle-income countries. In Malaysia, common symptoms observed in both outpatients and inpatients include fatigue, brain fog, depression, anxiety, insomnia, and joint or muscle pain (12). However, in India, a single-center prospective observational cohort study highlighted fatigue, dyspnea, and weight loss as the predominant symptoms among hospitalized patients (13). A study in China found that at 6-month follow-up, fatigue or muscle weakness and sleep difficulties were the main symptoms observed in COVID-19 patients who had recovered. Patients with more severe illness had reduced lung function and a higher risk of psychological complications like anxiety and depression (14). In line with these findings and following similar results in high-income countries (2, 3, 15, 16), our study identified fatigue and memory loss as the most prevalent symptoms among both hospitalized and non-hospitalized patients. These results underscore the wide range of symptoms and the potential impact on various patient populations affected by long COVID.

Previous literatures showed that symptoms due to long COVID have a strong impact on quality of life of affected patients (17, 18). While our study found no difference in the EQ-VAS visual analog scale between patients with and without long COVID at 90 days compared to 30 days after COVID-19, the long COVID group exhibited a lower quality of life score according to the EuroQol-5D3L questionnaire. This contrasts with findings from a high-income country, where a Japanese report indicated that participants with long COVID had lower average scores on both the EQ-VAS and EuroQol-5D3L compared to those without long COVID (19). Gaspar et al. (20) in a study conducted in Portugal showed an association between the presence of long COVID and the deterioration of quality of life, assessed through the EQ-5D index, at 3-, 6-, and 9-months post-discharge. Further research is needed to better understand the long-term effects of COVID-19, including how it affects the quality of life of those with persistent symptoms.

Previous studies, including those conducted in low-and middle-income countries, have established a link between female gender and long COVID (12, 13, 21–23), However, it has not been previously reported that a positive depression screening at day 30 using a validated questionnaire could be a risk factor for diagnosing long COVID at day 90 (24, 25). Additionally, few studies have examined depression as a risk factor for COVID-19 or long COVID (26, 27). Taquet et al. showed a bidirectional association between COVID-19 and psychiatric disorder. Adults with a history of COVID-19 diagnosis have an approximately doubled risk of being newly diagnosed with a psychiatric condition than those without SARS-CoV-2 infection. On the other hand, having a diagnosis of psychiatric disorder in the year before the COVID-19 pandemic was associated with a 65% increased risk of COVID-19 (28). More recently, an investigation of factors associated with psychiatric outcomes in long COVID was published and it was shown that patients with long COVID are at increased risk for psychiatric disease, including depression, compared with those without long COVID (29). Conversely, Wang et al. found a strong association between symptoms of depression and anxiety, worry about COVID-19, loneliness, and perceived stress with the risk of long COVID. They note that their results should not be misinterpreted as being supportive of the hypothesis that symptoms of long COVID are psychosomatic since a significant number of patients without mental illness also develop long COVID (30).

The association between depression and long COVID may be explained by several factors. Inflammation and activation of the hypothalamic–pituitary–adrenal axis, which can lead to chronic immune suppression, can be generated by distress. Additionally, depression may lead to changes in the brain and nervous system, which could contribute to long COVID symptoms such as fatigue and cognitive impairment (31). This suggests that recommending mental health screening to support these patients might be warranted. The PHQ-2 is a simple and effective screening tool that can be used to assess depression symptoms. Early identification and treatment of depression may help prevent the development of long COVID and improve overall health outcomes.

While hypertension is known to increase the risk of severe COVID-19 illness, the underlying mechanisms are not fully understood (32). Hypertension may contribute to the development of long COVID by affecting cardiovascular health and immune function. Initially, there was a suggested link between the use of renin-angiotensin-aldosterone system (RAAS) inhibitors and mortality in severe SARS-CoV-2 infection due to interactions with the bradykinin pathway. However, subsequent evidence has not confirmed this hypothesis (33–37). Other studies have indicated that hypertension’s association with known risk factors such as advanced age, obesity, diabetes, cardiovascular disease, and chronic kidney disease could explain its role as a risk factor (38, 39). Limited research has been conducted on the role of hypertension as a predictor of long COVID and the impact of RAAS inhibitors on long-term symptoms. A recent study involving 414 patients indicated that hypertension appears to play a significant role in the persistence of long COVID symptoms. Among these individuals, 39.6% reported symptoms extending beyond 6 weeks post-infection. The study found that long COVID was notably higher in patients over 65 years old and those with various comorbidities, including Type II diabetes mellitus, dyslipidemia, coronary artery disease, asthma, and cancer. Specifically, hypertension showed an odds ratio of 2.59 and was statistically significant (*p* = 0.001), indicating a notable association with prolonged symptoms post-infection (40).

Similar to a study conducted in Italy, our results indicate that the severity of acute COVID-19 (ICU admission, prolonged length of stay, and use of mechanical ventilation) did not exert a substantial influence on the development of long-term COVID-19 (41). Interestingly, our findings showed that patients who received steroids during hospitalization were at greater risk of developing this condition. Likewise, a study from Southeastern Italy also demonstrated that corticosteroid therapy administered in the acute phase of COVID-19 might be associated with an increased risk of long COVID. One plausible hypothesis posited by the authors is that the administration of corticosteroids during the acute phase of illness may potentially contribute to the persistence of the virus within non-respiratory system among some patients reservoirs (24). To ascertain the potential association between corticosteroid utilization and an increased risk of prolonged COVID-19, along with its dependence on factors such as dosage, type, or duration of in-hospital steroid therapy, further comprehensive investigations are warranted.

The Omicron era was found to be associated with a lower risk of developing long COVID among hospitalized patients while age ≥ 60 years old was a protective factor among non-hospitalized patients, similar to that reported by Reme et al. (42). The findings of a study published in 2022 demonstrated that the mean number of post-COVID-19 symptoms was higher in patients infected with the Wuhan variant than in those infected with the Alpha or Delta variant (43). Meanwhile a correspondence published in the same year found a reduction in the odds of long COVID with the Omicron variant versus the Delta variant (44). This intriguing finding suggests that the emergence of different COVID-19 variants may influence the clinical course and outcomes of this disease, including the likelihood of experiencing long-term symptoms. Understanding the role of the new COVID-19 variants in long COVID may aid in tailoring treatment approaches and public health interventions to mitigate its long-term burden. This study has several strengths. Its innovative approach focuses on exploring long COVID within the context of a middle-income country, specifically in Brazil. By comparing occurrences and predictive factors between hospitalized and non-hospitalized patients, the study provides crucial insights into how the severity of the initial illness influences the manifestation and impact of long COVID on individuals. Moreover, the study delves into the use of patient-reported outcome measures and their long-term effects on conditions such as COVID, examining their influence on both quality of life and mental health.

Our study sheds light on the prevalence of long COVID in distinct patient groups and identifies potential predictors, including gender, underlying health conditions, and depression screening. These findings offer crucial information for comprehending, predicting, and managing long COVID in diverse patient cohorts. By focusing on a middle-income country, the research contributes to a more comprehensive understanding of long COVID by incorporating data from varied socioeconomic backgrounds.

### 4.1 Limitations

This study has limitations due to its single institution setting and limited timeframe. The generalizability of the findings to broader populations may be limited. The reliance on clinical data and lack of exploration of certain factors, such as pre-existing conditions, especially presence of depression or mood disorder, may contribute to variations in outcomes and the prevalence of long COVID. Variables related to comorbidities were collected from the medical records; however, the Charlson Comorbidity Index was not routinely measured, so we did not use this information in our analysis. Socioeconomic data were not collected for this study. Additionally, the study’s retrospective nature and reliance on self-reported symptoms introduce biases and variability in reporting. Furthermore, the study did not thoroughly examine the impact of treatment regimens other than remdesivir, or vaccination status. Due to the sensitive nature of personal information within vaccination data, access to this information in national databases is limited under the General Data Protection Law established in 2020 in Brazil. Consequently, these data was not included in the analyses. As of July 2022, at the conclusion of the Omicron wave, approximately 90% of the population of the State of São Paulo had received the first dose of the COVID-19 vaccine. We believe this fact may have contributed to the protective outcome during the Omicron wave in the multivariate analysis. Moreover, our study did not specifically address the characteristics of patients diagnosed with the COVID variants (Alpha/Gamma, Delta, and Omicron). Given the evolving nature of the virus and the emergence of new variants, it is essential to recognize that the dynamics of long COVID may be influenced by factors specifically to each variant. Therefore, extrapolation of our findings to populations affected by more recent variants should be approached with caution, and further research is warranted to understand the implications of these variants on the manifestation and impact of long COVID.

Future research with larger cohorts and prospective designs is needed to validate these findings, explore the underlying mechanisms, and address these limitations for a more comprehensive understanding of long COVID.

## 5 Conclusion

In conclusion, approximately half of both hospitalized and non-hospitalized patients in Brazil developed long COVID 90 days after their initial COVID-19. The prevalence of long COVID differed among different strain eras, with fatigue and memory loss being the most frequently reported symptoms. We identified a significant association between a positive depression screening at day 30 and an increased risk of developing long COVID at day 90. These findings may highlight the importance of integrating depression screening into regular COVID-19 follow-ups at primary care clinics.

## Data availability statement

The raw data supporting the conclusions of this article will be made available by the authors, without undue reservation.

## Ethics statement

The studies involving humans were approved by Ethics Committee of Hospital Israelita Albert Einstein – CEP/Einstein. The studies were conducted in accordance with the local legislation and institutional requirements. The ethics committee/institutional review board waived the requirement of written informed consent for participation from the participants or the participants’ legal guardians/next of kin because the waiver of the use of informed consent is based on: i) being a retrospective observational study that utilized only medical records, institutional information systems, and/or other sources of data and clinical information available within the institution without the use of biological material anticipated; ii) because all data were handled and analyzed anonymously, without nominal participant identification; iii) because the study results will be presented in an aggregated manner, preventing individual participant identification; and iv) because it is a non-interventional study (without clinical interventions) and without alterations/influences in the participant’s routine/treatment, consequently posing no additional risks or harm to their well-being. Additionally, we will be unable to obtain consent from all participants in this research.

## Data sharing agreement

De-identified individual study data reported in this article can be requested upon application to investigator board via the corresponding author.

## Author contributions

DMa: Conceptualization, Formal analysis, Methodology, Supervision, Writing – review & editing. SB-P: Conceptualization, Investigation, Methodology, Writing – original draft, Writing – review & editing. KP: Writing – original draft, Methodology, Formal analysis. JP: Conceptualization, Investigation, Writing – original draft. EP: Data curation, Formal analysis, Validation, Visualization, Writing – original draft. DMe: Data curation, Methodology, Project administration, Supervision, Writing – original draft. CA: Data curation, Project administration, Writing – original draft. BP: Data curation, Writing – original draft. VL: Writing – original draft. SA: Data curation, Validation, Writing – original draft. PT: Writing – review & editing. CL: Writing – review & editing. MN: Writing – review & editing. SK: Writing – review & editing. VT: Conceptualization, Resources, Writing – review & editing. TK: Writing – review & editing. ME: Writing – review & editing. AM: Conceptualization, Investigation, Methodology, Supervision, Writing – review & editing.

## Footnotes

^1^ https://www.knime.com/knime-analytics-platform

^2^ https://www.r-project.org/
