# Supplementary material for: Prevalence, predictors, and patient-reported outcomes of long COVID in hospitalized and non-hospitalized patients from the city of São Paulo, Brazil
Source: Front Public Health. 2024 Jan 22;11:1302669. doi: 10.3389/fpubh.2023.1302669 (PMC10839020; doi:10.3389/fpubh.2023.1302669)
Supplement: Supplementary file 1 [file Table_1.DOCX]

Supplementary Material

# Supplementary Data

**Supplementary data 1.** R PACKAGE

TABLES, STATISTICAL TESTS AND UNIVARIATE REGRESSION -> gtsummary (https://www.danieldsjoberg.com/gtsummary/)

DATA IMPORTING, TRANSFORMATION, MANIPULATION AND VISUALIZATION -> tidyverse (https://www.tidyverse.org/)

MULTIVARIATE

REGRESSION -> stats (https://www.rdocumentation.org/packages/stats/versions/3.6.2/topics/glm)

VIF EVALUATION -> car (https://rdrr.io/cran/car/man/vif.html)

NORMALITY TEST -> nortest (<https://cran.r-project.org/web/packages/nortest/index.html>)

# Supplementary Figures and Tables

## Supplementary Tables

| Characteristic | Overall  N = 291 | Alpha/Gamma  N = 151 | Delta  N = 15 | Omicron  N = 125 | p-value*^*^* |
| --- | --- | --- | --- | --- | --- |
| ICU admission, n(%)*^†^* | 123(42.27%) | 63(41.72%) | 13(86.67%) | 47(37.60%) | **<0.01** |
| Mechanical ventilation, n(%) | 28(9.62%) | 22(14.57%) | 0(0.00%) | 6(4.80%) | **0.01** |
| Antibiotics, n(%) | 173(59.45%) | 98(64.90%) | 12(80.00%) | 63(50.40%) | **0.01** |
| Corticosteroids, n(%) | 203(69.76%) | 141(93.38%) | 6(40.00%) | 56(44.80%) | **<0.01** |
| Remdesivir, n(%) | 36(12.37%) | 13(8.61%) | 2(13.33%) | 21(16.80%) | 0.10 |
| *^*^* Pearson’s Chi-squared test; Fisher’s exact test *^†^* ICU = Intensive Care Unit | | | | | |

**Supplementary Table 1.** COVID-19 management for hospitalized patients

## Supplementary Figure
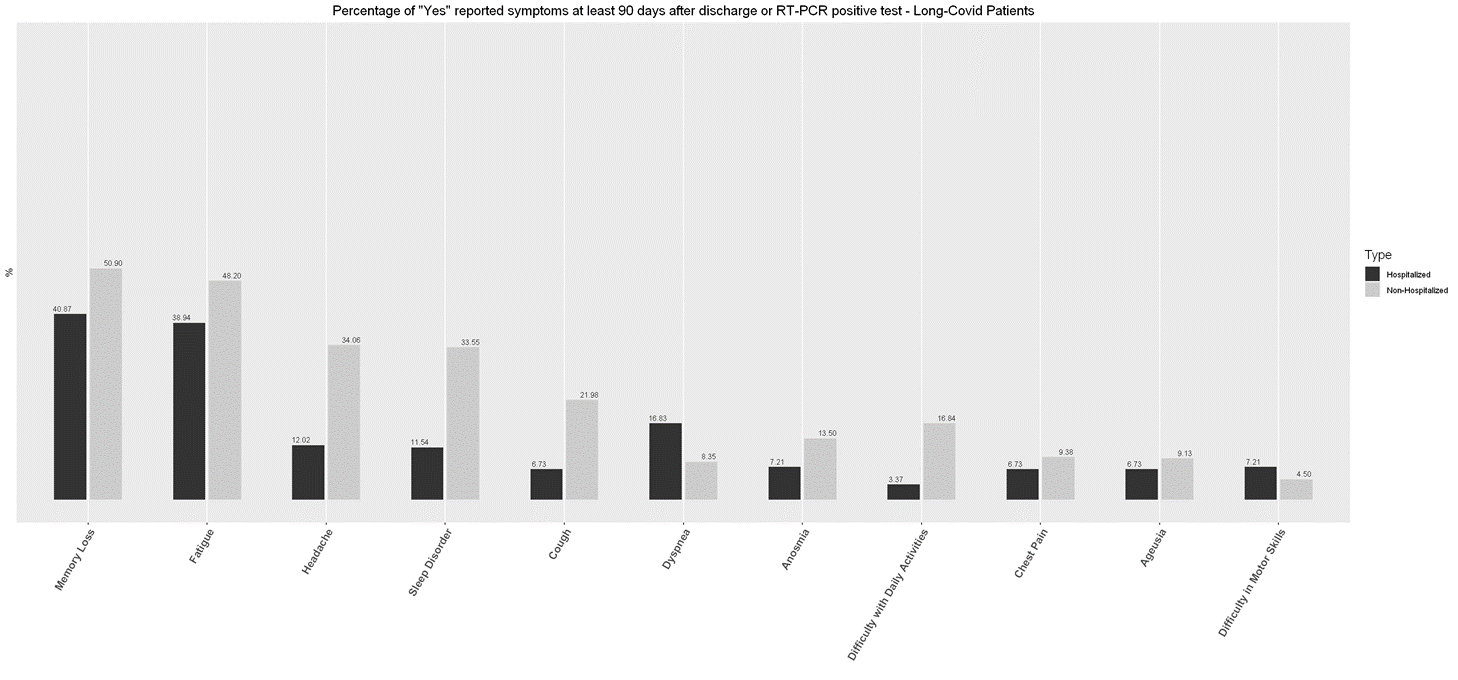
Supplementary Figure 1. Distribution of 90 day-symptom of Long COVID patients by studied groups (hospitalized and non-hospitalized)


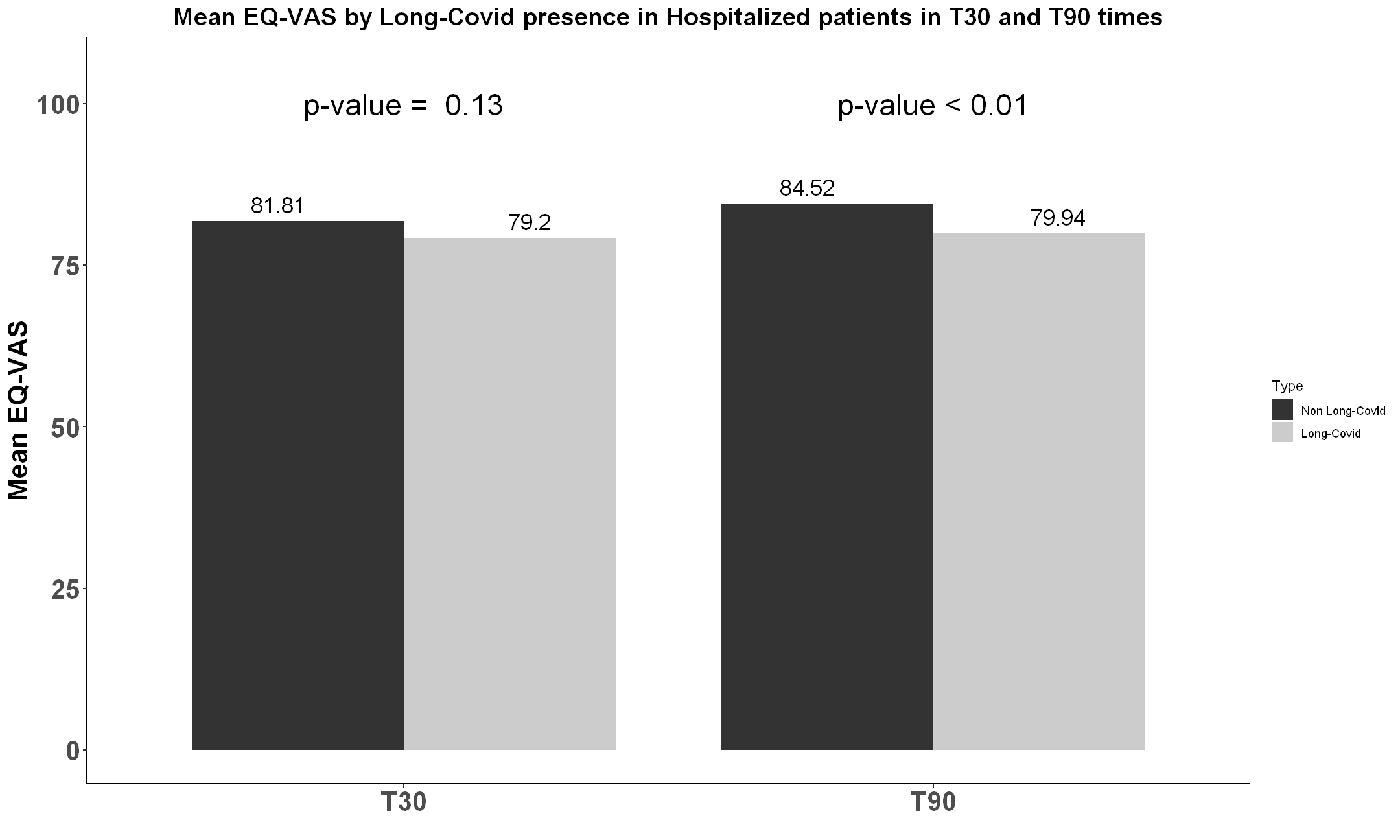


**Supplementary Figure 2.** Mean EQ-VAS by long COVID presence in hospitalized patients in T30 and T90 times

**
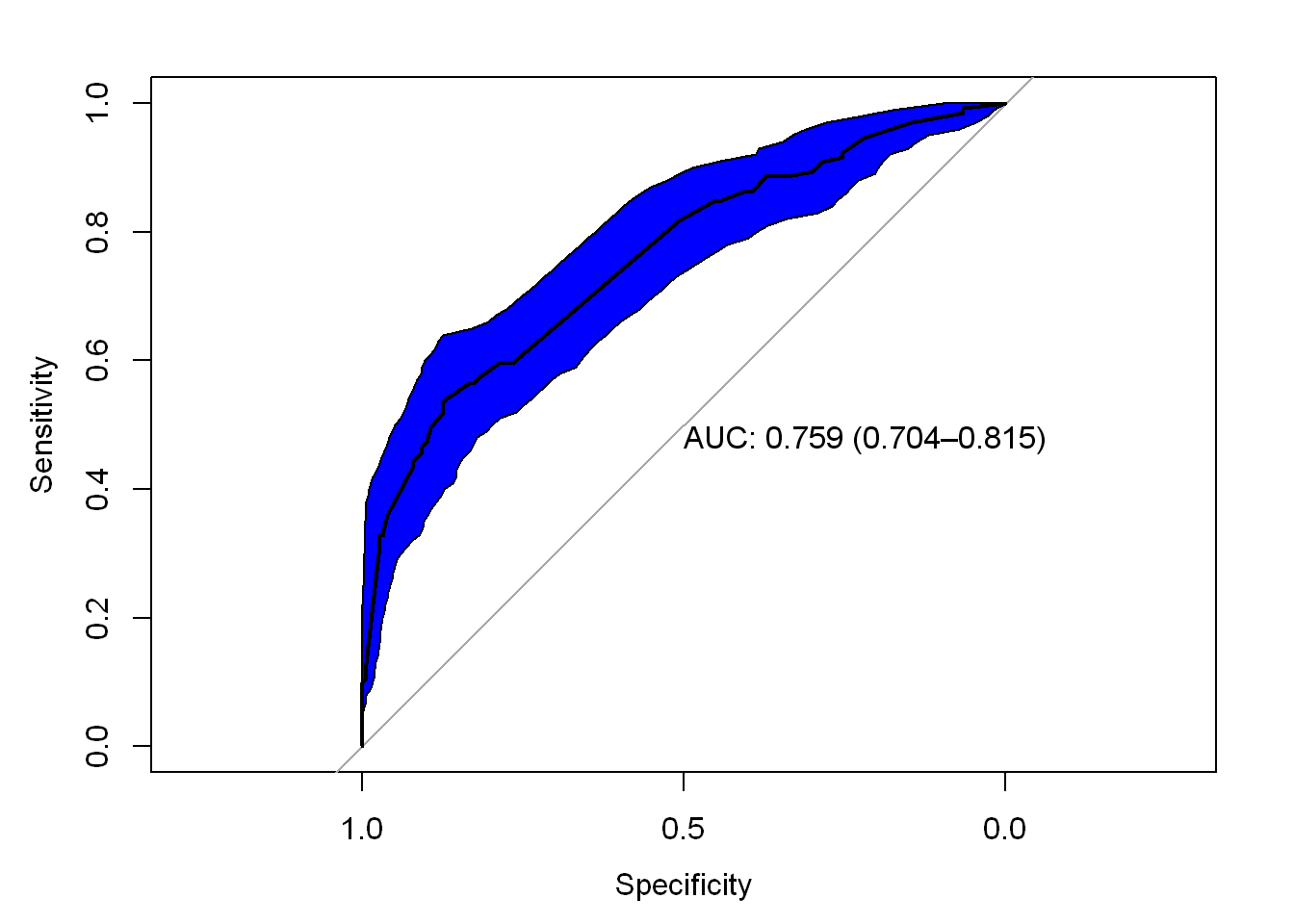
**

**Supplementary Figure 3.** Receiver operating characteristic (ROC) analysis– Hospitalized Group


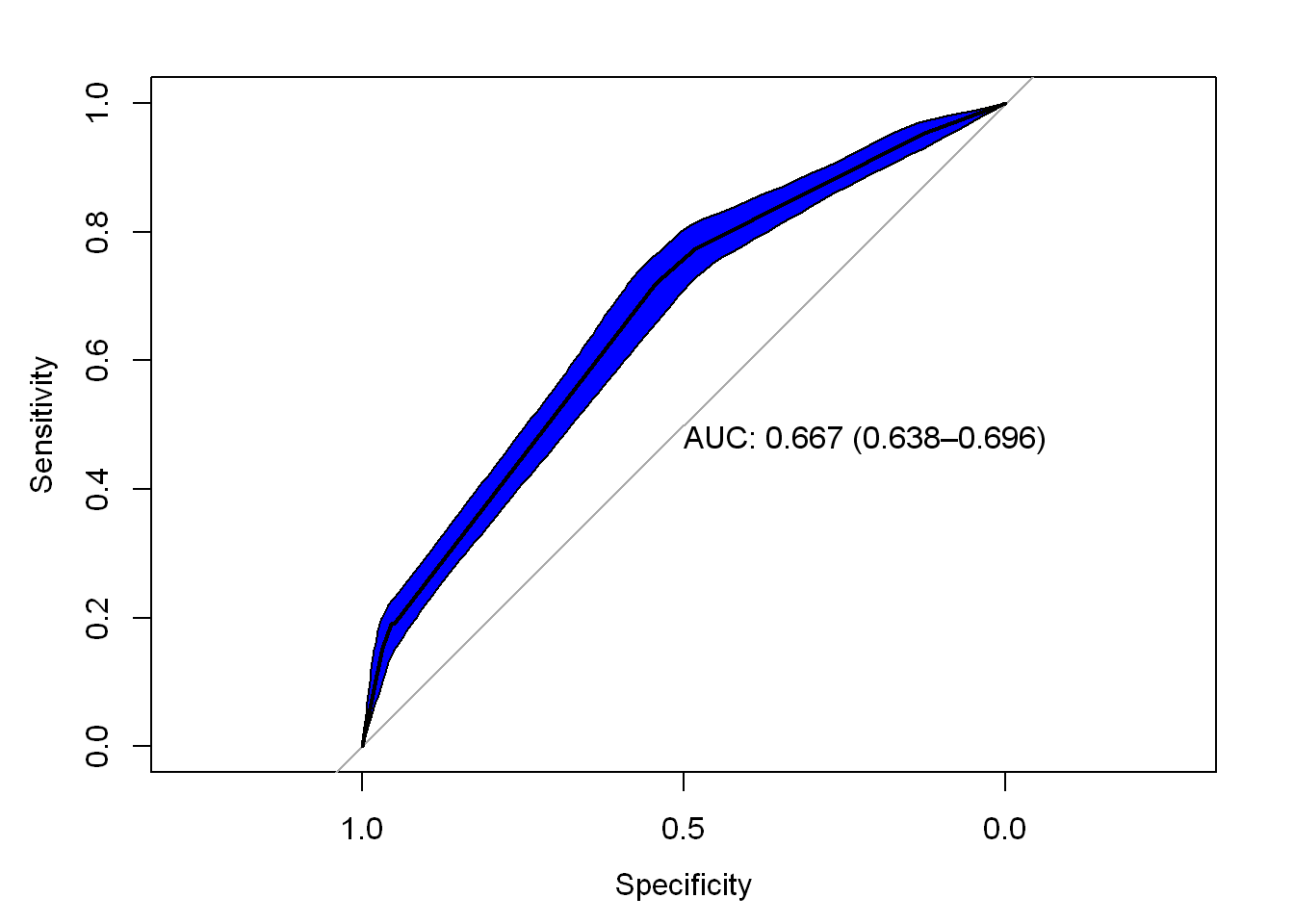


**Supplementary Figure 4.** Receiver operating characteristic (ROC) analysis–Non- hospitalized Group
